# Associations of birth mode with cord blood cytokines, white blood cells, and newborn intestinal bifidobacteria

**DOI:** 10.1371/journal.pone.0205962

**Published:** 2018-11-02

**Authors:** Isabel Cristina Ribas Werlang, Noel Theodore Mueller, Aline Pizoni, Henrique Wisintainer, Ursula Matte, Sergio Hofmeister de Almeida Martins Costa, Jose Geraldo Lopes Ramos, Marcelo Zubaran Goldani, Maria Gloria Dominguez-Bello, Helena Ayako Sueno Goldani

**Affiliations:** 1 Laboratory of Translational Pediatrics, Hospital de Clínicas de Porto Alegre, Universidade Federal do Rio Grande do Sul. Porto Alegre–RS, Brazil; 2 Post-Graduate Program in Health of Child and Adolescent, Faculdade de Medicina, Universidade Federal do Rio Grande do Sul. Porto Alegre–RS, Brazil; 3 Department of Epidemiology, Johns Hopkins University Bloomberg School of Public Health, Baltimore, Maryland, United States of America; 4 Welch Center for Epidemiology, Prevention, and Clinical Research, Johns Hopkins Medical Institutions, Baltimore, Maryland, United States of America; 5 Post-Graduate Program Sciences in Gastroenterology and Hepatology, Faculdade de Medicina, Universidade Federal do Rio Grande do Sul. Porto Alegre–RS, Brazil; 6 Universidade Luterana do Brasil—ULBRA, Porto Alegre–RS, Brazil; 7 Department of Genetics, Universidade Federal do Rio Grande do Sul. Porto Alegre–RS, Brazil; 8 Department of Gynecology and Obstetrics, Hospital de Clínicas de Porto Alegre, Universidade Federal do Rio Grande do Sul and Hospital Mae de Deus. Porto Alegre–RS, Brazil; 9 Department of Biochemistry and Microbiology and of Anthropology, Rutgers University, New Brunswick, New Jersey, United States of America; Columbia University, UNITED STATES

## Abstract

The associations of Cesarean delivery with offspring metabolic and immune-mediated diseases are believed to derive from lack of mother-to-newborn transmission of specific microbes at birth. *Bifidobacterium spp*., in particular, has been hypothesized to play a health-promoting role, yet little is known about how delivery mode modifies colonization of the newborn by this group of microbes. The aim of this research was to examine the presence of *Bifidobacterium* in meconium and in the transitional stool, and to assess cytokine levels and hematological parameters in the venous cord blood of infants born by elective, pre-labor Cesarean section vs. vaginal delivery in Southern Brazil. We recruited 89 mother-newborn pairs (23 vaginal delivery and 66 elective cesarean delivery), obtained demographic information from a structured questionnaire and clinical information from medical records. We obtained umbilical cord venous blood and meconium samples following delivery and the transitional stool (the first defecation after meconium) before discharge. We determined plasma levels of IL-1β, IL-10, IL-6, GM-CSF, IL-5, IFN-γ, TNF-α, IL-2, IL-4 and IL-8 in the cord blood, and presence of stool *Bifidobacterium* by real time PCR. Compared to vaginally-delivered neonates, Cesarean-delivered neonates had a lower leukocyte count (*p* = 0.037), lower hemoglobin (*p* = 0.04), and lower levels of the cytokine GM-CSF (*p* = 0.009) in the cord blood. Moreover, *Bifidobacterium* was detected less often in the transitional stool of Cesarean-delivered neonates compared to vaginally-delivered neonates (*p* = 0.001). The results indicate that pre-labor Cesarean birth may be associated with microbial and hematological alterations in the neonate. The clinical significance of these findings remains to be determined in larger prospective birth cohort studies.

## Introduction

The prevalence of Cesarean delivery is increasing in many parts of the world, including most notably in Brazil, where, according to the Brazilian Ministry of Health, the rate has risen to over 50% [1]. The rising prevalence is most likely due to a rise in elective Cesarean deliveries [1]. Due to the ubiquity of Cesarean deliveries worldwide, understanding the health consequences of this unnatural medical intervention has become increasingly important to public health.

A growing number of studies have found that Cesarean-delivered children and adults are at higher risk for a range of metabolic and immune-mediated diseases compared to their vaginally-delivered counterparts. Our group was the first to report a link between Cesarean delivery and offspring obesity in Brazil [2]. This finding has since been corroborated in other populations [3–6], and was confirmed by a large prospective study that included siblings [7]. Yet, the mechanism underlying the higher risk of obesity among Cesarean-delivered offspring remains unclear.

The leading mechanistic hypothesis is that differential newborn acquisition of specific maternal microbiota at birth alters infant microbiome development along with immune and metabolic programming [8–9]. In particular, *Bifidobacterium spp*., which are seeded early in life and break down oligosaccharides in human breast milk [8] may be differentially acquired at birth. *Bifidobacterium spp*. have also been associated with obesity [10–11], raising the possibility that delayed colonization by this anaerobe after birth may play a role in the future development of obesity.

It has also been postulated that differential stress response to labor may, in part, contribute to the higher risk of obesity in Cesarean-delivered offspring [12]. Labor in vaginal delivery is a stressful experience that has been associated with higher levels of various fetal leukocytes, including monocytes [13] and granulocytes [14–16]. During labor, the activation of cytokines has also been found in systemic maternal circulation [17–19]. To the best of our knowledge, however, no studies have compared multiple cytokines and hematologic parameters in cord blood from vaginally-deliveries vs. pre-labor Cesarean deliveries. Understanding whether cord blood cytokines and hematologic factors are associated with delivery mode will help inform the design and interpretation of future studies on delivery mode, the infant microbiome and risk of obesity and other health outcomes.

The aim of this study was to examine the presence of *Bifidobacterium* in meconium and in the transitional stool (defecation after meconium), and to assess cytokine levels and hematologic parameters in the cord blood of infants born by pre-labor Cesarean delivery vs. vaginal delivery in Southern Brazil. We hypothesized that compared to vaginal delivery, Cesarean delivery is associated with lower cord-blood leukocyte counts and lower prevalence of *Bifidobacterium* in neonatal stool.

## Materials and methods

### Design and population

In 2011, we recruited 89 mother-newborn pairs (23 had vaginal delivery and 66 pre-labor Cesarean delivery) from a private hospital in Porto Alegre, Brazil. We invited mothers delivering between 38 and 42 weeks (confirmed by ultrasonography performed before the 20^th^ week of gestational age) to participate. We enrolled mothers after written informed consent. We excluded women with HIV/AIDS, preeclampsia, diabetes, hypertension, autoimmune disorders, and women who smoked, were on a restrictive diet, or took antibiotics in the third trimester. Mothers agreed to provide umbilical cord blood, meconium, and the stool following meconium (i.e. transitional stool) from their newborn. They also agreed to complete a postnatal questionnaire adapted for this study [20]. For vaginal births, we only included women whose water broke <12 hours before delivery. Our study was approved by the Research and Ethics Committee of the Hospital de Clinicas (protocol no. 11/0388) and the Hospital Mae de Deus (protocol no. 524/11) located in Porto Alegre, Brazil.

**Fig 1:**
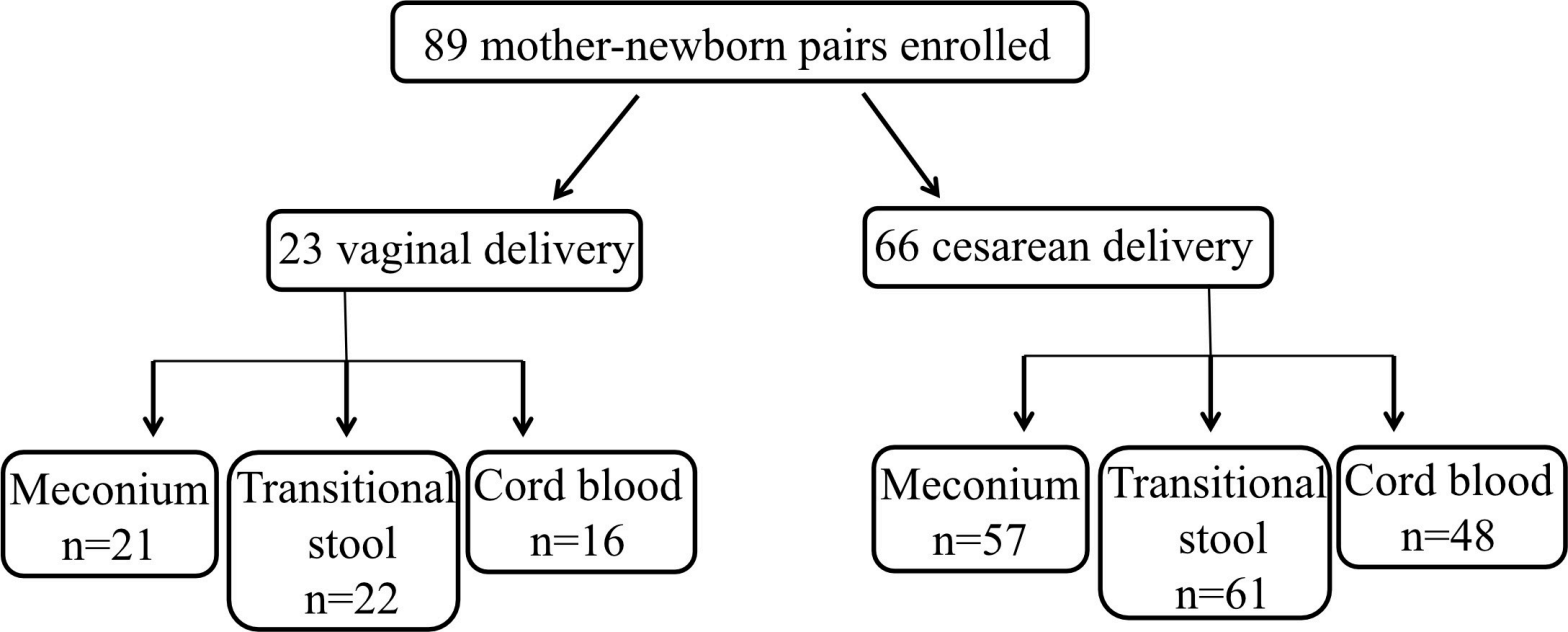
Flow diagram of the number of participants that contributed to various analyses.

Out of 89 mother-infant pairs enrolled, we collected umbilical cord blood from 64, meconium from 78, and transitional stool from 85 (83 of which were analyzed for this study as 2 stool samples were accidentally lost during specimen strorage and transport) (Fig 1). We were not able to collect meconium from the neonates who defecated prior to collection nor were we able to collect transitional stools from those who did not defecate during the inpatient period.

### Assessment of participant characteristics

We extracted clinical information from medical records and an investigator administered postnatal questionnaire. Specifically, we collected information on mode of delivery, gravidity, parity, history of urinary tract infection during pregnancy, antibiotic use during pregnancy, gestational age, birth weight, and birth length from medical records. Pre-pregnancy weight (kg) and height (m) were self-reported on the postnatal questionnaire. Body mass index (BMI) was calculated as weight in kilograms divided by height squared (kg/m^2^).

### Venous cord blood and measurement of leukocytes and cytokines

We collected umbilical cord venous blood (10 mL) with EDTA tubes according to the local routine protocols. We stored 4 mL at 4°C until processing within 12 hours for complete blood count. Cell counts were performed by photometry, cytochemistry and impedance techniques using a Sysmex XE 5000 hematology-analyzer in the clinical laboratory of Hospital de Clínicas de Porto Alegre. We immediately centrifuged the remaining blood (6 mL) for 10 minutes at 2000 rpm to separate plasma, which was then stored at -80°C until cytokines analysis assessment.

We determined plasma levels of IL-1β, IL-10, IL-6, GM-CSF, IL-5, IFN-γ, TNF-α, IL-2, IL-4 and IL-8 in the umbilical cord venous blood by use of Human Ultrasensitive Cytokine Magnetic 10-Plex Panel (Life Technologies) according to the manufacturer’s instructions in a Luminex 200 equipment with the xPONENT 3.1 software. We assayed samples in duplicate and the detection limits (pg/mL) of the cytokines were: 0.05 for IL-1β, IL-10, GM-CSF and TNF-α, 0.1 for IL-6, IL-5, IL-2 and IL-8, 0.5 for IFN-γ and IL-4.

### Fecal collection, DNA extraction and measurement of *Bifidobacterium*

We collected approximately 5 grams of both meconium and transitional stool from the diapers with sterile spatulas. We placed these specimens in sterile tubes at 4°C for up to 6 hours and then stored them at -80°C until processing. The stool collection was scheduled every 3 hours according to the local routine care.

We carried out DNA extraction using QIAamp DNA Stool minikit (QIAGEN) according to the instructions provided by the manufacturer. Following the extraction (200 μL of final volume), we concentrated samples to 20 μL using 3M Na Acetate pH 5.2 and stored them at -20°C until analysis. We determined the DNA concentration using the NanoDrop 1000 Spectrophotometer (Thermo Scientific, Life Technologies). A stool sample of a healthy child of 3 months of age was used as a positive control for the DNA extraction protocol and the detection of the amplicons by real-time PCR.

To detect *Bifidobacterium spp*., we analyzed, by real-time PCR using TaqMan System (Applied Biosystems, Life Technologies), 78 samples of meconium DNA (21 from vaginally-delivered neonates and 57 from Cesarean-delivered neonates) and 83 samples of transitional stool DNA (22 from vaginally-delivered neonates and 61 from Cesarean-delivered neonates) from the 89 mother-infant dyads consented and enrolled in the study. Primers and probe (sequence 5’→3) included were Bifido F (GCG TGC TTA ACA CAT GCA AGT C), Bifido R (CAC CCG TTT CCA GGA GCT ATT) and BifidoProbe [(VIC) TCA CGC ATT ACT CAC CCG TTC GCC–(BHQ-1)] [21]. We conducted real-time detections of the amplicons in a total volume of 15 μL containing 1 X TaqMan universal PCR Master mix, 2 μM of both primers, 2.5 μM of probe and 4.5 μL of purified DNA. We performed the amplification (2 minutes at 50°C, 10 minutes at 95°C, and 40 cycles of 15 seconds at 95°C and 1 minute at 60°C) and detection using a Stratagene Mx3000P detection system (Agilent Technologies). We used purified DNA from *Bifidobacterium breve* (ATCC) to detect bifidobacteria. We expressed samples as positive for bifidobacteria if they had a cycle threshold ≤ 30.1 (≈ 2 pg/mL of specific DNA) and negative for those with cycle threshold > 30.2.

### Statistical analyses

We calculated the mean and standard deviation of normally distributed characteristics and the median and 25^th^ and 75^th^ percentiles of non-normally-distributed variables. We tested for differences in participant characteristics according to delivery mode using parametric or non-parametric statistical tests depending on the distribution of data, which we identified using the Kolmogorov–Smirnov test. We used unpaired *t* tests to compare unpaired normally-distributed data and Mann-Whitney *U* tests for unpaired data that did not follow normal distribution. We used Fisher exact test to compare proportions. We computed 95% confidence intervals and a two-sided *p* < 0.05 was considered significant.

## Results

Of the 89 mother-newborn dyads that met study inclusion criteria, 23 underwent vaginal delivery and 66 Cesarean delivery. Demographic data of all participants are presented in Table 1 and those with cytokine measurements are presented in S1 Table. Compared to mothers that delivered vaginally, mothers that delivered by Cesarean section had higher pre-pregnancy BMI, but otherwise there were no significant differences in the clinical characteristics of the mother or her infant between the two groups (Table 1).

**Table 1:** Characteristics of all participants.

	Total (n = 89)	Vaginal Delivery (n = 23)	Cesarean Delivery (n = 66)	*p* value*
Birth weight, *g*, mean (± SD)	3249.6 (380.7)	3192.6 (274.2)	3269.5 (411.3)	0.32
Birth length, *cm*, mean (± SD)	48.9 (1.7)	48.7 (1.8)	48.9 (1.6)	0.66
Gestational age, *weeks*, mean (± SD)	38.8 (0.8)	39.0 (0.9)	38.7 (0.7)	0.06
No. prenatal visits, mean (± SD)	9.3 (1.5)	9.7 (1.3)	9.2 (1.6)	0.13
Prepregnancy BMI, *kg/m*^*2*^, mean (± SD)	24.5 (4.3)	22.9 (3.5)	25.1 (4.5)	0.045
Gestational wt gain, *kg*, mean (± SD)	13.7 (5.0)	12.1 (3.6)	14.2 (5.4)	0.11
Mother’s age, *years*, mean (± SD)	30.1 (5.1)	29.7 (6.1)	30.3 (4.7)	0.63
Parity, median (25th-75th %ile)	2.0 (1.0–2.0)	2.0 (1.0–2.0)	1.5 (1.0–2.0)	0.08

*based on Student’s *t* test

In a subsample of participants (n = 28), we measured hematological factors in umbilical cord venous blood. Leukocyte count was lower in the cord blood of Cesarean deliveries (n = 21) compared to vaginal deliveries (n = 7) [mean (SD): 9.99 (1.99) x 10^3^/μL vs. 13.55 (3.52) x 10^3^/μL, respectively, *p* = 0.037]. Hemoglobin was also significantly lower in cord blood from Cesarean deliveries compared to vaginal deliveries [mean (SD): 15.20 (1.54) g/dL vs. 16.22 (1.96) g/dL, respectively, *p* = 0.04] (Table 2). There also appeared to be differences in red blood cell count and hematocrit (%), but these differences did not reach statistical significance with our sample size (*p* values of 0.16 and 0.06, respectively).

**Table 2:** Hematologic parameters of venous cord blood according to type of delivery.

	Total (n = 28)	Vaginal Delivery (n = 7)	Cesarean Delivery (n = 21)	*p* value*
White cells count (x 10^3^/μL)^a^	10.9 (2.9)	13.6 (3.5)	10.0 (2.0)	0.037
Red cells count (x 10^3^/μL)^a^	4.3 (0.5)	4.5 (0.4)	4.2 (0.5)	0.16
Hemoglobin (g/dL)^a^	15.2 (1.5)	16.2 (2.0)	14.9 (1.3)	0.04
Hematocrit (%)^a^	45.4 (4.7)	48.2 (5.6)	44.4 (4.1)	0.06

^a^values presented as mean (±SD)

*based on unpaired *t* test

Among the 10 cytokines examined, only GM-CSF was significantly lower in the venous cord blood of Cesarean deliveries compared to vaginal deliveries [Median (25th-75th %ile): 0.18 (0.09–0.23) *vs* 0.27 (0.17–0.34) pg/mL, *p* = 0.009]. This finding remained even after adjusting for gestational age (*p* = 0.006). There were no significant differences in the other cytokines according to delivery mode (Table 3).

**Table 3:** Venous cord blood cytokine levels in 16 vaginal deliveries and 48 Cesarean deliveries.

	Vaginal Delivery (n = 16)	Cesarean Delivery (n = 48)	
	Median (25th-75th %ile)	Median (25th-75th %ile)	*p* value*
GM-CSF (pg/mL)	0.27 (0.17–0.34)	0.18 (0.09–0.23)	0.009
IFN Gamma (pg/mL)	1.80 (0.63–2.76)	2.01 (0.63–2.68)	0.95
IL1 Beta (pg/mL)	1.21 (0.86–2.05)	1.31 (0.97–2.15)	0.69
IL2 (pg/mL)	0.28 (0.20–0.37)	0.14 (0.00–0.30)	0.08
IL4 (pg/mL)	2.49 (2.13–2.68)	1.84 (0.88–3.55)	0.20
IL5 (pg/mL)	0.35 (0.27–0.51)	0.44 (0.26–0.73)	0.23
IL6 (pg/mL)	2.78 (2.42–3.83)	2.47 (1.73–3.37)	0.11
IL8 (pg/mL)	25.66 (14.44–58.15)	20.74 (14.18–40.87)	0.52
IL10 (pg/mL)	1.24 (1.00–1.58)	1.15 (1.01–1.57)	0.76
TNF Alpha (pg/mL)	1.72 (1.20–2.11)	1.44 (0.82–2.08)	0.36

*based on Mann-Whitney *U* Test

DNA concentration was significantly lower in the meconium and transitional stool of Cesarean-delivered neonates compared to vaginally-delivered neonates (Table 4). There was no evidence of *Bifidobacterium* in the meconium samples of either group. In the transitional stool, *Bifidobacterium* was detected in 11 of 83 samples (13.3%). *Bifidobacterium* was found less often in stools from Cesarean-delivered neonates (positive in 3 of 61 samples; 4.9%) compared to vaginally-delivered neonates (positive in 8 of 22 samples; 36.4%) (*p* = 0.001) (Table 5). We examined and did not find an association of pre-pregnancy BMI with presence of bifidobacteria, cytokines levels, or blood markers (data not shown), indicating that the association between delivery mode and these factors is unlikely to be confounded by maternal weight status.

**Table 4:** DNA concentration in meconium and transitional stool according to type of birth.

	Vaginal Delivery		Cesarean Delivery		
	*n*	DNA concentration (ng/μL) ^a^	*n*	DNA concentration (ng/μL) ^a^	*p* value*
Meconium	21	3.3 (1.9–5.8)	57	2.0 (1.0–3.8)	0.03
Transitional stool (ng/μL)	22	10.8 (7.1–28.0)	61	5.5 (4.2–9.0)	<0.001

^a^Median (25th-75th %ile)

*based on Mann-Whitney *U* Test

**Table 5:** *Bifidobacteria* DNA detected by real time PCR in meconium and transitional stool according to type of birth.

	Vaginal Delivery		Cesarean Delivery		
	*n*	*Bifidobacteria* DNA detected, *n (%)*	*n*	*Bifidobacteria* DNA detected, *n (%)*	*p* value*
Meconium	21	0 (0)	57	0 (0)	-
Transitional stool	22	8 (36.4)	61	3 (4.9)	0.001

*based on Fisher exact test

## Discussion

In our convenience sample of Brazilian mothers and their newborns, neonates born by pre-labor Cesarean delivery had a lower prevalence of *Bifidobacterium* and total DNA in their transitional stool compared to neonates born by vaginal delivery. Cesarean delivery was also associated with lower levels of venous cord blood leukocytes, hemoglobin, and granulocyte-macrophage colony-stimulating factor (GM-CSF), which has been shown to influence leucocyte count in pregnancy [22].

Our finding of differences in prevalence of *Bifidobacterium* and DNA concentration may be driven by the fact that vaginally-delivered babies acquire a rich inoculum of vaginal and intestinal microbiota from their mother during newborn passage through the birth canal. Neonates delivered by pre-labor Cesarean section, on the other hand, miss out on this microbially-rich bath. High-throughput amplicon sequencing of bacterial DNA has shown that vaginally-delivered infants are colonized with organisms mainly from the mother's vaginal and intestinal microbiota, whereas Cesarean-delivered infants are colonized with skin and environmental microbiota [23–25]. Our findings are also consistent with other studies that have shown that compared to vaginally-delivered infants, Cesarean-delivered infants intestinal flora is characterized by a notable absence of *Bifidobacterium spp*. [24,26–27].

We cannot rule out the possibility that delayed initiation of breastfeeding, which has been observed after Cesarean delivery [28–29], might also contribute the differences in *Bifidobacterium* prevalence by delivery mode in our study. There are more than 200 oligosaccharide structures found in human breast milk that are fermented and made into useable energy by *Bifidobacterium spp*. Human breast milk may also directly provide the infant with *Bifidobacterium* [30–31]. Future studies are needed to test this hypothesis.

Our cytokine findings contribute to a small number of studies that have explored and found cytokine differences in the cord blood of vaginal deliveries compared to Cesarean deliveries [12]. These differences could contribute to postnatal immune system development [12,32–33]. To the best of our knowledge, we are the first study to find that cytokine GM-CSF was lower in the venous cord blood of Cesarean deliveries. We also confirmed previous studies that showed higher leukocytes counts in the cord blood of vaginal deliveries compared to Cesarean deliveries [13–16,34]. Together, our findings of GM-CSF and leukocytes have biologic plausibility [12], as GM-CSF has been shown to influence leucocyte counts in pregnancy [22]. The association of GM-CSF and other hematologic parameters with offspring metabolic health warrants future research.

A strength of this study is that it was carried out in a unique sample of mother-newborn pairs, including mothers that underwent elective, pre-labor Cesarean delivery. Yet, one major limitation was the small sample size of our study. We cannot rule out the possibility that some of our null findings would have been significant with a larger sample, nor can we rule out that some of our significant findings were due to chance. Our small sample size also precluded us from building statistically-stable, multivariable-adjusted regression models. Instead, we tried to minimize confounding in our study design by excluding mothers who delivered prematurely, used antibiotics in the third trimester, had an extreme diet pattern during pregnancy, or had existing infections or chronic diseases. We also examined and did not find an association of pre-pregnancy BMI with presence of *Bifidobacterium*, cytokines levels, or hematologic parameters, leading us to believe that the association between delivery mode and these factors were unlikely to be confounded by maternal weight status. Nevertheless, we cannot rule out the possibility that some of the associations observed in our study were due to factors other than delivery mode (e.g. feeding).

In conclusion, Cesarean delivery was associated with lower cord blood levels of the cytokine GM-CSF, lower cord-blood leukocyte counts, and lower prevalence of *Bifidobacterium* in the transitional stool of neonates. Given the high prevalence of Cesarean delivery worldwide, there is need for larger prospective studies to determine the potential long-term clinical significance of our findings.

## Supporting information

S1 Table Characteristics of participants with cytokine data available. (DOC)

S1 File Study_DATASET.sav: Database with the information used for the study. (SAV)
