# Supplementary material for: Associations of birth mode with cord blood cytokines, white blood cells, and newborn intestinal bifidobacteria
Source: PLoS One. 2018 Nov 2;13(11):e0205962. doi: 10.1371/journal.pone.0205962 (PMC6214518; doi:10.1371/journal.pone.0205962)
Supplement: S1 Table — (DOC) [file pone.0205962.s001.doc]

**Supporting information**

**S1Table. Characteristics of participants with cytokine data available.**

|  | Total (n=64) | Vaginal (n=16) | Cesarean (n=48) | *p^a^* |
| --- | --- | --- | --- | --- |
| Birth weight, *g*, mean (± SD) | 3248.5 (390.9) | 3144.0 (306.9) | 3283.4 (412.2) | 0.16 |
| Birth length, *cm*, mean (± SD) | 48.8 (1.7) | 48.5 (2.1) | 48.9 (1,5) | 0.43 |
| Gestational age, *weeks*, mean (± SD) | 38.7 (0.7) | 38.9 (0.9) | 38.3 (0.7) | 0.25 |
| No. prenatal visits, mean (± SD) | 9.3 (1.5) | 9.6 (1.4) | 9.3 (1.5) | 0.51 |
| Prepregnancy BMI, *kg/m^2^*, mean (± SD) | 24.5 (4.7) | 23.0 (3.9) | 25.0 (4.9) | 0.13 |
| Gestational wt gain, *kg*, mean (± SD) | 13.5 (4.4) | 12.9 (2.7) | 13.7 (4.8) | 0.52 |
| Mother’s age, *years*, mean (± SD) | 30.0 (4.8) | 28.2 (5.9) | 30.7 (4.3) | 0.07 |
| Gestations, median (25th-75th%ile) | 2.0 (1.0-2.0) | 2.0 (1.2-3.5) | 2.0 (1.0-2.0) | 0.15 |

^a^Student’s T-Test
